# Sensitivity Enhanced Ecofriendly UV Spectrophotometric Methods for Quality Control of Telmisartan and Benidipine Formulations: Comparison of Whiteness and Greenness with HPLC Methods

**DOI:** 10.3390/ijerph19127260

**Published:** 2022-06-14

**Authors:** Muhammad Shahzad Chohan, Mahesh Attimarad, Katharigatta Narayanaswamy Venugopala, Anroop Balachandran Nair, Nagaraja Sreeharsha, Efren II Plaza Molina, Ramling Bhagavantrao Kotnal, Sheeba Shafi, Marysheela David, Pottathil Shinu, Abdulrahman Ibrahim Altaysan, Abdulmalek Ahmed Balgoname

**Affiliations:** 1 Department of Biomedical Sciences, College of Clinical Pharmacy, King Faisal University, Al-Hofuf 31982, Al-Ahsa, Saudi Arabia; mshwhan@kfu.edu.sa (M.S.C.); spottathail@kfu.edu.sa (P.S.); 2 Department of Pharmaceutical Sciences, College of Clinical Pharmacy, King Faisal University, Al-Hofuf 31982, Al-Ahsa, Saudi Arabia; kvenugopala@kfu.edu.sa (K.N.V.); anair@kfu.edu.sa (A.B.N.); sharsha@kfu.edu.sa (N.S.); aaltaysan@kfu.edu.sa (A.I.A.); abalgoname@kfu.edu.sa (A.A.B.); 3 Department of Biotechnology and Food Science, Faculty of Applied Sciences, Durban University of Technology, Durban 4000, South Africa; 4 Department of Pharmaceutics, Vidya Siri College of Pharmacy, Off Sarjapura Road, Bangalore 560035, India; 5 Department of Pharmacy Practice, College of Clinical Pharmacy, King Faisal University, Al-Hofuf 31982, Al-Ahsa, Saudi Arabia; emolina@kfu.edu.sa; 6 Department of Pharmaceutical Chemistry, BLDEs College of Pharmacy and Research Centre, Vijayapur 586103, India; rbkotnal@gmail.com; 7 Department of Nursing, College of Applied Medical Sciences, King Faisal University, Al-Hofuf 31982, Al-Ahsa, Saudi Arabia; sheeba@kfu.edu.sa (S.S.); mdavid@kfu.edu.sa (M.D.)

**Keywords:** benidipine, formulation, quantification, ratio derivative spectroscopy, scaling factor, telmisartan, validation

## Abstract

The development of an environmentally friendly analytical technique for simultaneous measurement of medicines with large concentration differences is difficult yet critical for environmental protection. Hence, in this work, new manipulated UV-spectroscopic methods with high scaling factors were established for concurrent quantification of telmisartan (TEL) and benidipine (BEN) in fixed-dose combinations. Two different methods were developed and established by calculation of peak height at zero crossing point of second derivative and the ratio of first derivative spectra with a scaling factor of 200 and 100, respectively. The absorption difference between the peaks and troughs of the ratio spectra, as well as continuous subtraction from ratio spectra, were established as additional methods. In addition, new procedures were validated using ICH recommendations. The proposed methods’ linearity curves were constructed in the range of 0.5–10 µg mL^−1^ and 1–30 µg mL^−1^ for BEN and TEL, respectively, under optimized conditions. Furthermore, both the detection (0.088–0.139 µg mL^−1^ for BEN and 0.256–0.288 µg mL^−1^ for TEL) and quantification limits (0.293–0.465 µg mL^−1^ for BEN and 0.801–0.962 µg mL^−1^ for TEL) were adequate for quantifying both analytes in the formulation ratios. The accuracy and precision were confirmed by the good recovery percent (98.37%–100.6%), with low percent relative error (0.67%–1.70%) and less than 2 percent relative standard deviation, respectively. The specificity of the methods was proven by accurate and precise outcomes from the standard addition method and analysis of laboratory mixed solutions with large differences in concentrations of both analytes. Finally, the BEN and TEL content of the formulations was determined simultaneously without prior separation using these first ever reported spectroscopic methods. Furthermore, developed UV derivative spectroscopic methods demonstrated high greenness and whiteness when compared to the reported HPLC methods. These findings show that the projected methods were effective, practical, and environmentally acceptable for quality control of BEN and TEL in multicomponent formulations.

## 1. Introduction

**Figure 1:**
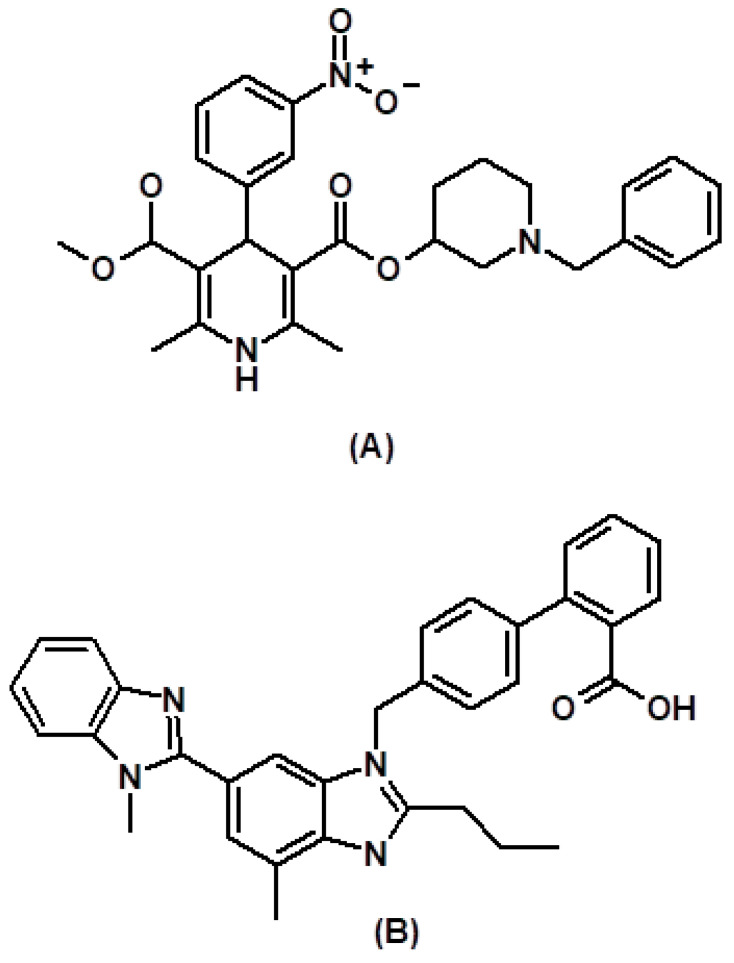
Chemical structure of benidipine (**A**) and telmisartan (**B**).

The prevalence of hypertension in middle and old age people is increasing and 1300 million people suffer from high BP worldwide. High blood pressure is responsible for 8.5 million deaths due to cardiovascular complications and kidney-related problems [1,2]. Hence, controlling normal blood pressure is essential. Different studies demonstrated that combination therapy with drugs having a different mechanism of action [3,4] helps in effective and rapid control of blood pressure. Benidipine and telmisartan are the recommended combinations of calcium channel blockers (CCBs) with angiotensin receptor blockers (ARBs) for the management of hypertension [5] because of their anti-proteinuric effects [6]. Benidipine (BEN, Figure 1A), a strong and long-acting calcium channel blocker, acts by inhibiting three subtypes of calcium channels (L, N, and T) and showed a renal protective effect [7]. It also showed a cardio protective effect due to increased nitric oxide production with better vascular selectivity [8]. Telmisartan (TEL, Figure 1B), an azole class angiotensin II receptor antagonist, acts by inhibiting the secretion of aldosterone by reversibly binding angiotensin II to the AT1 receptor present on vascular smooth muscle and adrenal glands. Thereby arterial blood pressure is decreased by decreasing the systemic vascular resistance. Telmisartan also showed PPAR-γ agonistic effect, which has beneficial effects on carbohydrate metabolism and antidiabetic property [9,10,11]. 

A number of assay procedures are reported in the literature for the estimation of benidipine and telmisartan alone in formulations and plasma. Benidipine alone, and in combination with other drugs, were estimated by UV-Vis spectrophotometric methods [12,13,14,15], derivative spectroscopic method [16], spectrofluorometric method [17], HPLC [18,19], and LC-MS/MS [20,21]. Analysis of telmisartan using spectrophotometric [22,23], HPLC [24,25,26,27,28], HPTLC [28], LC-MS/MS [29,30], and capillary electrophoresis [31] was reported in the literature. Many HPLC procedures are depicted for the assay of benidipine and telmisartan in pharmaceutical preparation [32,33]. For simultaneous quantification of benidipine and telmisartan, Naim M et al. reported stability—indicating the HPLC approach [34]. Patel B. et al. [35] reported simultaneous determination of TEL and BEN along with chlorthalidone by RP-HPLC. Further, the HPLC method requires expensive instrumentation and uses toxic chemicals such as methanol and acetonitrile. However, to date, no spectrophotometric method has been reported for concurrent quantification of benidipine and telmisartan in the formulation. The objective of the present work was to develop green, sensitive, and simple spectrophotometric methods, which can replace the expensive, hazardous chromatographic methods for the white analysis of TEL and BEN in bulk and fixed-dose combinations. However, the complete overlap of UV absorption spectra and the wide difference in the amount of the two drugs in the formulation makes it difficult to establish the spectrophotometric procedure for concurrent quantification without prior separation. Derivative spectroscopic methods allow quantification of compounds having complete overlapping spectra by computing the peak height at the zero-crossing point. The ratio derivative spectroscopic method removes the effect of one of the components by dividing the combined spectra of analytes with the spectrum of another component [36,37,38,39,40,41,42,43]. Hence, derivative and ratio derivative spectrophotometric methods were developed in the present work. However, developing an analytical method for real-time measurement of components with huge concentration variations is challenging. To boost the sensitivity of low concentration medicines in the formulation, a large scaling factor was applied. Furthermore, to conserve the environment, the development of a green analytical approach is now needed. As a result, recently created quantitative greenness and whiteness evaluation methods were used to calculate the environmental sustainability of the established methods and compared with the reported HPLC methods. Finally, for simultaneous quantification of benidipine and telmisartan in laboratory mixed solutions and fixed-dose formulation, established modified UV spectroscopic methods were used.

## 2. Materials and Methods

Analytically pure standards of benidipine and telmisartan were procured from Biokemix India Limited (Hyderabad, India). Analytical grade ethanol was procured from Sigma Aldrich (Darmstadt, Germany). High purity water utilized for preparing the solutions was prepared using a Milli Q water purifier (Milli Pore, Burlington, MA, USA). The dosage forms consisting of benidipine 4 mg with telmisartan 40 mg and 80 mg were obtained from the local pharmacy. UV-Vis spectrophotometer (Shimadzu 1650, Japan) linked to a personal computer was used. Quartz cuvettes with a path length of 10 mm were used for both blank and standard. UV absorption spectra were generated by scanning at high speed with a slit width of 1 nm and smoothened after manipulation using 10 nm, wherever necessary. UV Probe software (Ver 2.0, Shimadzu, Japan) was used to scan and manipulate the UV absorption spectra. 

### 2.1. Preparation of Standard Solutions and Laboratory Mixed Solution

TEL and BEN (1 mg mL^−1^) standard solutions were produced separately by putting 100 mg of TEL into a volumetric flask (100 mL) containing ethanol and 100 mg of benidipine hydrobromide in 100 mL of water. Working standards and validation solutions were also arranged by adding dilute ethanol (10% *v*/*v*) to the stock solution. By transferring the required volume of standard solutions and diluting them with water, laboratory mixed solutions containing various ratios of BEN and TEL were made.

### 2.2. Procedure for Each Method

#### 2.2.1. Second Derivative Method (SDM)

The required amounts of BEN and TEL known-concentrations solutions were placed separately into a 10 mL volumetric flask to obtain concentrations of 0.5–10 g mL^−1^ and 1–24 g mL^−1^, respectively. UV absorption spectra were recorded and stored in the computer for all of the solutions in the wavelength range of 200–400 nm, using a 10 percent *v*/*v* ethanol solution as a blank. The zero-order spectra were transformed to second-order spectra using the scaling factor of 200 and a wavelength of 10 nm. The peak amplitude 2nd derivative spectra of BEN at varying concentrations were recorded at 239.8 nm. Similarly, the peak amplitude 2nd derivative spectra of TEL at varying concentrations were recorded at 233.1 nm. The peak amplitude was plotted against corresponding BEN and TEL concentrations to create the calibration curves for BEN and TEL, respectively. Further, regression equations and coefficients were generated. 

#### 2.2.2. Ratio Amplitude Difference Method (RAD)

UV absorption spectra were generated in the wavelength range of 200–400 nm using working standard BEN and TEL solutions of 4 µg mL^−1^ and 5 µg mL^−1^, correspondingly. An appropriate amount of BEN and TEL stock standard solutions were placed into 10 mL graduated flasks for the preparation of six solutions with concentrations of 0.5–10 µg mL^−1^ and 1–30 µg mL^−1^, correspondingly. UV absorption was measured for all of the solutions in the region of 200–400 nm. The ratio spectra for BEN were generated by dividing the aforesaid absorption spectra by the spectrum of TEL (5 µg mL^−1^) and smoothed with ∆λ = 10 nm before being saved. By deducting the peak height at 244.5 nm from the peak amplitude at 305.4 nm, the peak height difference was obtained. The linearity curve was then created by graphing the difference in peak amplitude against the relevant concentration. Similarly, the ratio spectra of TEL were generated by dividing the recorded mixed spectra by the BEN (4 µg mL^−1^) spectra. The calibration curve was constructed by deducting the peak height at 304.2 nm from the peak height at 360.8 nm at various concentrations. 

#### 2.2.3. Ratio First Derivative Method (RFD)

The above-recorded ratio spectra for BEN were transformed into first derivative spectra with a scaling factor of 100, using 10 nm as the reference wavelength (∆λ). The linearity curve was created by graphing the peak height at 318.6 nm against the concentration of BEN. Similarly, TEL ratio spectra were transformed to first derivative spectra using a scaling factor of 100 and 10 nm as ∆λ. The linearity curve was created by graphing the peak amplitude at 344 nm against the respective TEL concentrations.

#### 2.2.4. Constant Subtraction Method (CSM)

A constant absorption value at 375 nm, sufficient to fetch the spectra to the starting position, was removed from the ratio spectra of TEL in the range of 1–30 g mL^−1^, and the resulting spectra were multiplied with the spectrum of BEN (4 µg mL^−1^). The absorbance was measured at 300 nm using the TEL zero spectra, and the linearity curve was built against respective concentrations, along with the regression equation and coefficient. Similarly, from the ratio spectra of BEN in the range of 0.5–10 g mL^−1^, a constant absorption at 303 nm was removed, and the resulting spectra were multiplied with the spectrum of TEN (5 µg mL^−1^). The calibration curve was made by graphing the absorbance at 238.2 nm from the generated zero-order BEN spectra against the corresponding BEN concentration.

#### 2.2.5. Procedure for Laboratory Mixed Solutions

The laboratory mixed solutions were organized by adding the required amount of BEN and TEL stock solutions slightly below and above the formulation concentration and within the linearity range, to obtain the ratios of 1:20, 5:20, 10:10, 0.5:5, and 2:24 µg mL^−1^, respectively. The laboratory mixed solutions were scanned in the wavelength range of 200–400 nm and the concentration of each analyte was determined following the general procedure of all four methods.

### 2.3. Procedure for Pharmaceutical Dosage Form

A marketed pharmaceutical preparation consisting of BEN (4 mg) with TEL (40 mg) and TEL (80 mg) was weighed separately and the mean weight was computed. Twenty tablets of each type were powdered separately and the triturate corresponding to 4 mg of BEN and 40 mg of TEL was dissolved in 50 mL of ethanol by sonicating for 15 min. The solution was filtered into another 100 mL graduated flask, the remainder was splashed with additional ethanol, and the absolute volume was attuned to 100 mL with ethanol. Similarly, a solution comprising 4 mg of BEN and 80 mg of TEL was prepared. The required amount of ethanol was added to maintain the 10% *v*/*v* ethanol in each solution. Furthermore, adequate quantities of water were mixed to bring the analytes’ concentrations within the linearity range and scanned in the wavelength range of 200–400 nm. Finally, the concentration of each analyte was determined following the general procedure of all four methods using concerned regression equations.

## 3. Results and Discussion

**Figure 2:**
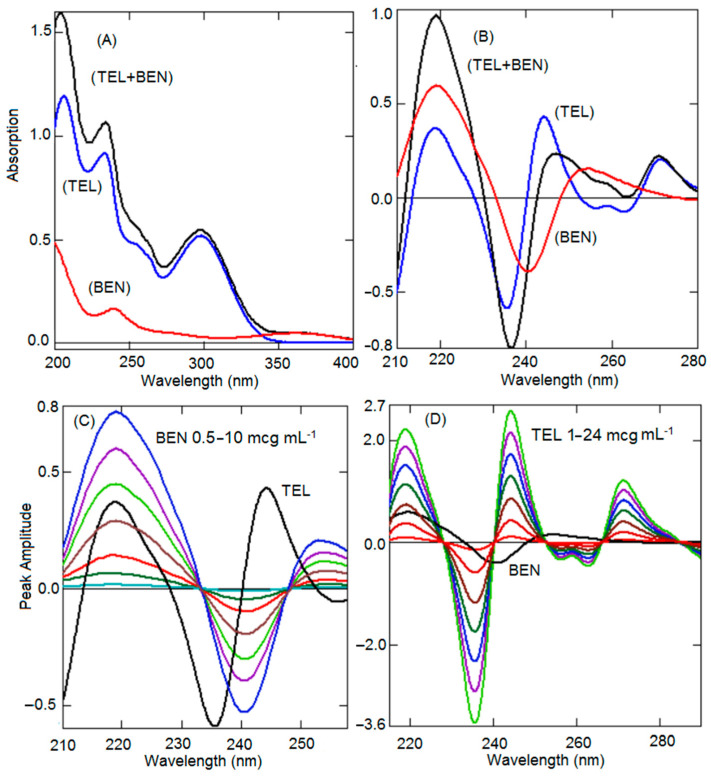
Zero-order UV absorption spectra of BEN (Red), TEL (Blue), and mixture (Black) (**A**). Second-order derivative spectra of BEN (Red), TEL (Blue), and mixture (Black) (**B**). Second-order derivative spectra of BEN (0.5–10 µg mL^−1^) (**C**). Second-order derivative spectra of TEL (1–24 µg mL^−1^) (**D**).

UV spectroscopic methods are extensively used for the quality control of drugs and are included in the official monographs for qualitative and quantitative analysis. Many scientific literatures reported that UV spectroscopic methods showed similar analysis results as HPLC methods [44,45]. Further, the UV spectroscopic method has limitations in terms of specificity, because impurities and degradation products cannot be quantified. However, inference from another analyte and formulation excipients can be removed by mathematically processed UV-spectroscopic method. In addition, it has many advantages such as rapid analysis, low operating cost, and low generation of waste. In the present work, both the analytes, TEL and BEN, showed good UV absorption, unfortunately, spectra showed complete overlap in the wavelength 200–350 nm, whereas BEN had some absorption above 350 nm and TEL had no absorption (Figure 2A). However, the straight UV absorption technique for the simultaneous quantification of both analytes is challenging without prior separation. Such binary mixtures can be quantified by eliminating interference from one another using possible modification procedures such as derivative, ratio spectroscopic, and ratio subtraction methods [36,37,38,39,40,41,42,43]. To increase the sensitivity of the low concentration, the BEN scaling factor was optimized. Further, to develop an ecofriendly analytical method, a safe solvent system was selected. BEN and TEL are slightly soluble in water but highly soluble in ethanol, hence 10% ethanol-water was utilized as a solvent system. 

### 3.1. Second Derivative Method (SDM)

The derivative spectroscopic method is extensively used for the analysis of formulations consisting of two or three analytes showing completely overlapped spectra (Figure 1A). Derivatization of UV spectra intensifies the specificity and selectivity along with the elimination of interference by other drug and formulation excipients. Further, measurement of absorption at a zero-crossing wavelength of one of the components was performed, at which additional compound having absorption allow quantification of one of the components without interference by another component. In the present work, second-order derivatization has been selected because first-order derivative spectra did not show any zero-crossing wavelengths where another component had some absorption. The formulation available in the market has a huge difference in the concentration of both the analytes. The amount of BEN is 10 and 20 times lower than the amount of TEL. A comparison of the first derivative spectra of BEN and TEL did not show any zero-crossing points for both analytes. The second derivative spectrum of BEN showed very low absorption compared to the TEL spectrum at zero-crossing points. To increase the absorption and sensitivity of low concentration analyte, different scaling factors were envisaged, starting from 10 to 200 times. With a scaling factor of 200 times, BEN was quantified at a very low concentration of 0.5 µg mL^−1^, hence a scaling factor of 200 was selected for further study. Further, a second-order derivative of BEN showed 233.1 nm, 247.7 nm, and 275.4 nm zero-crossing points where TEL had some absorption (Figure 2B). However, wavelength of 233.1 nm had high intensity and good sensitivity. A second derivative spectrum of TEL showed many zero-crossing points (212.3 nm, 228.0 nm, 239.8 nm, 256.2 nm, and 266.2 nm) at which BEN showed absorption (Figure 2B). On the other hand, at 239.8 nm the intensity of peak height was better and linear, hence 239.8 nm was selected for further study. Different concentrations of BEN (0.5–10 µg mL^−1^) and TEL (1–24 µg mL^−1^) solutions were scanned, changed into second derivative spectra using 10 nm as ∆λ, with a scaling factor of 200 (Figure 2C,D), and the peak heights were recorded at 239.8 nm and 233.1 nm for BEN and TEL. 

### 3.2. Ratio Amplitude Difference Method (RAD)

As per the Beer–Lambert law, the absorption of the multicomponent is described by Equation (1)

A_BT_ = Ԑ_B_C_B_ + Ԑ_T_C_T_ (1)

where A_BT_ is the combined absorption of BEN (B) and TEL (T), Ԑ_B_ and Ԑ_T_ are molar extinction coefficients, and the corresponding amounts of BEN and TEL are C_B_ and C_T_. When a mixture spectrum is divided by the spectrum of TEL at concentration T’ (A_T’_ = Ԑ_T’_C_T’_), ratio spectra for compound B are generated, which eliminate the influence of absorption of another component (T), as shown in Equation (2).

A_BT_/A_T’_ = A_B_/A_T’_ + C_T_/C_T’_ (2)

where C_T_/C_T’_ is constant (K), further simplification of Equation (2) by changing the ratio spectra of the mixture (A_BT_/A_T’_) with θ_A_ and A_B_/A_T’_ ratio spectra of only one analyte with θ_B_ leads to Equation (3).

θ_A_ = θ_B_ + K (3)

The constant K can be eliminated in different ways. One of the methods is the subtraction of peak amplitudes at two selected wavelengths (λ_1_ and λ_2_) as per the below equations. Generally, the two wavelengths selected were peak and trough to eliminate the noise.

∆θ = θ_A1_ − θ_A2_ = (θ_B1_ + K) − (θ_B2_ + K) = θ_B1_ − θ_B2_

where θ_B1_ and θ_B2_ are peak amplitude difference determined at peak and trough wavelengths λ_1_ and λ_2_, representing the peak height of single analyte B, and interference from another analyte (T) is ended. The ratio spectra of different concentrations of BEN were generated and the linearity curve was plotted by determining the peak amplitude difference between peak and trough against corresponding concentrations of BEN. 

**Figure 3:**
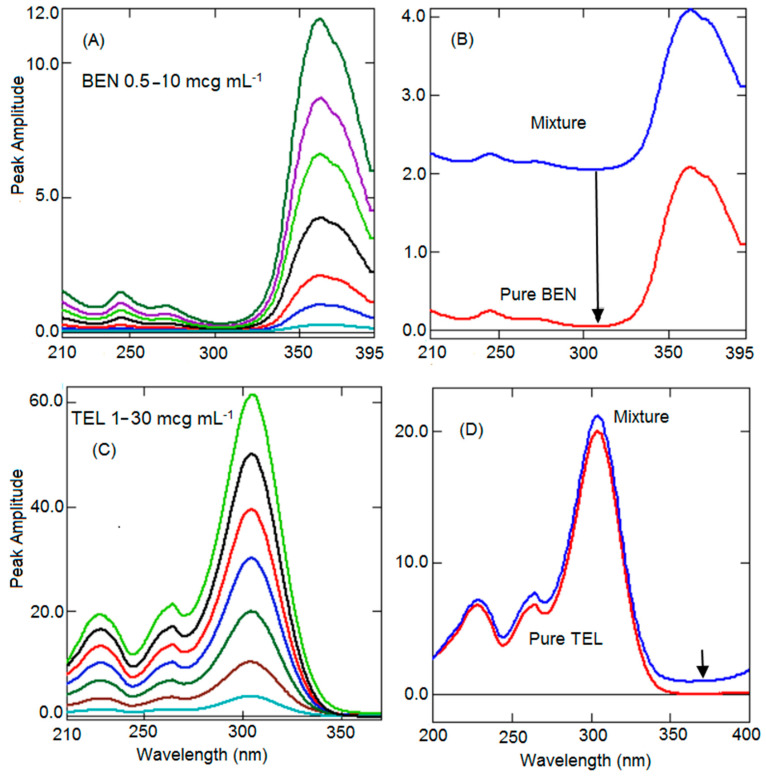
Ratio absorption spectra of BEN (**A**). Ratio absorption of BEN pure (Red) and mixture (Blue) (**B**). Ratio absorption spectra of TEL (**C**). Ratio absorption of TEL pure (Red) and mixture (Blue) (**D**). Arrow position indicates the wavelength at which constant was subtracted.

In the present work, ratio spectra of BEN and TEL were generated by dividing the series of BEN and TEL spectra by TEL (5 µg mL^−1^) and BEN (4 µg mL^−1^), respectively. The peak height difference between peak (305.4 nm) and trough (244.5 nm) was measured from the ratio spectra of BEN having different concentrations (Figure 3A), and plotted to generate a linearity curve for BEN. A similar peak height difference was observed in the ratio spectra generated from the mixture and the pure BEN spectra, confirming the elimination of interference of the TEL (Figure 3B). Similarly, a linearity curve was constructed for TEL by calculating peak height difference by measuring the peak heights at peak (360.8 nm) and trough (304.2 nm) from the series of ratio spectra of TEL (Figure 3C). Comparison of ratio spectra of the mixture and pure TEL (Figure 3D) showed the same height difference confirming the elimination of interference of the BEN.

### 3.3. Ratio First Derivative Method (RFD)

**Figure 4:**
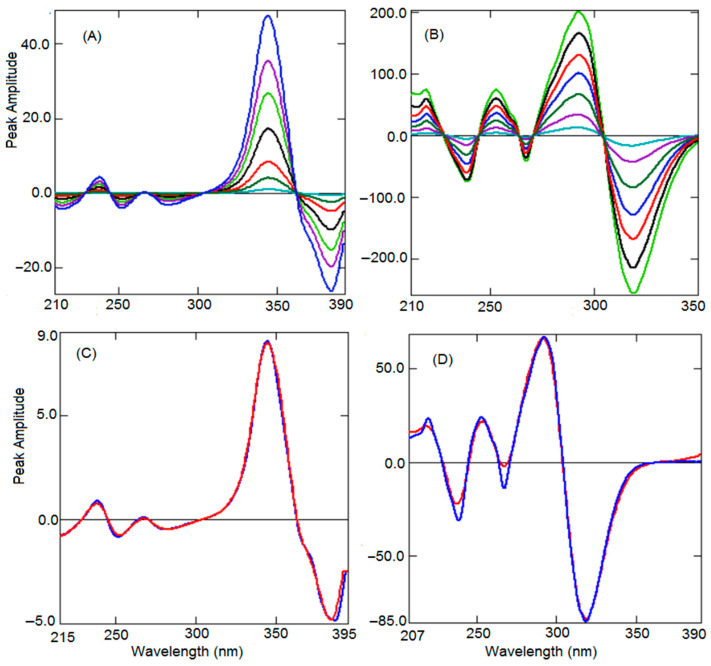
Ratio first derivative spectra of BEN (0.5–10 µg mL^−1^) (**A**). Ratio first derivative spectra of TEL (1–30 µg mL^−1^) (**B**). Comparison of ratio first derivative spectra of pure (Red) BEN (**C**) and TEL (**D**) with the mixture (Blue).

The derivatization of any number is zero, hence, from Equation (3), the constant (K) can be excluded by manipulating the ratio spectrum into the derivative spectrum. The derivative spectrum shows many maxima and minima, peak amplitude at a maxima or minima wavelength corresponds to only one analyte eliminating the effect of another analyte and excipients. This is a substitute to the above-explained ratio difference method to exclude the constant and quantify BEN in presence of TEL and vice versa. The first derivative spectra were produced from the series of ratio spectra of BEN using 10 nm as ∆λ along with the scaling factor of 100. Different wavelengths of 2–10 nm and different scaling factors of 10–200 were envisaged. A wavelength of 10 nm and a scaling factor of 100 produced good sensitivity. Two maxima at 238.0 nm and 344.0 nm along with four minima at 215.0, 252.0, 282.0, and 384.0 nm were observed in the first derivative spectra of BEN (Figure 4A). However, peak height was better at 344 nm with excellent sensitivity at low concentration. The recovery and linearity were also better at 344 nm; hence, 344.0 nm was used to prepare the linearity curve. Similarly, first derivative spectra for TEL were generated by adopting the 10 nm wavelength and scaling factor of 100 (Figure 4B). A wavelength of 318.6 nm was selected from the three maxima (219.6 nm, 253.0 nm, and 292.1 nm) and three minima (238.6 nm, 267.2 nm, and 318.6 nm) due to good peak height and recovery and linearity curves plotted using corresponding concentrations. Comparison of the first derivative spectra of pure BEN and TEL with the mixture spectra confirmed the exclusion of the influence of one of the analytes (Figure 4C,D). 

### 3.4. Constant Subtraction Method (CSM)

**Figure 5:**
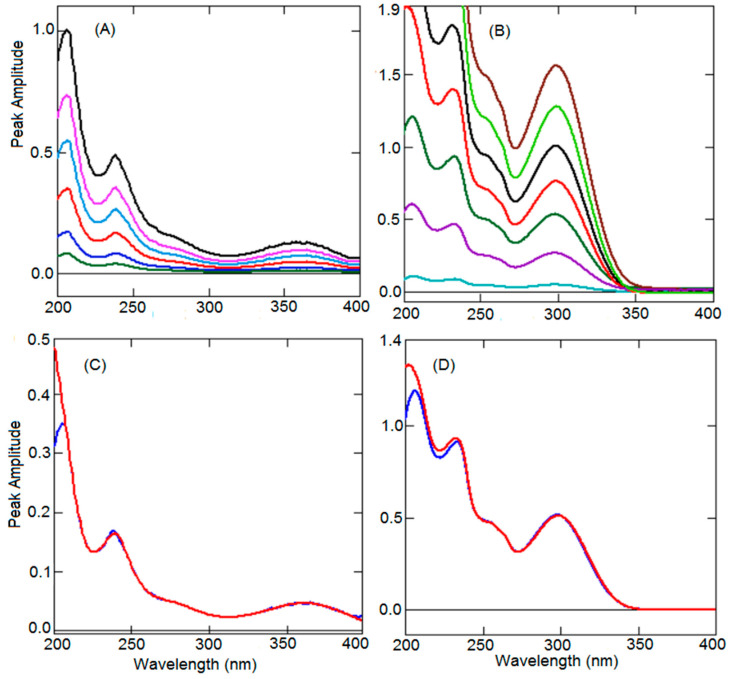
Zero-order spectra of BEN (0.5–10 µg mL^−1^) (**A**). Zero-order spectra of TEL (1–30 µg mL^−1^) (**B**). Comparison of zero-order spectra of pure (Red) BEN (**C**) and TEL (**D**) with the mixture (Blue).

The ratio absorption method depends upon the measurement of absorption at λ_max_ of zero-order UV absorption spectra of the analytes generated from the mixture spectra. From Equation (3), deduction of absorption value of constant (C_T_/C_T’_) from spectra ratio spectra of the mixture in upland are shown in Figure 3B,D to generate ratio spectra of pure BEN analyte, which eliminates the effect of TEL. The resulting ratio spectra were multiplied by the spectrum of TEL (5 µg mL^−1^) used as a divisor to produce the zero-order spectra of BEN (Figure 5A). The absorption was computed at 238.2 nm for different concentration spectra of BEN and a calibration graph was generated. Likewise, the normal spectrum of TEL (Figure 5B) was generated by subtraction of a constant absorbance and then multiplied with the spectrum of BEN (4 µg mL^−1^) used as devisor. The peak height of the series of TEL spectra was determined at 299.5 nm and plotted against respective concentrations of TEL. A comparison of generated zero-order spectra with the untreated pure analyte spectra produced at the same peak height confirms that one analyte is being quantified without the intervention of another (Figure 5C,D).

### 3.5. Method Validation

As per the requirement of the International Conference on Harmonization (ICH) guidelines, the developed analytic method was validated for linearity, the limit of detection and quantification, accuracy, precision, and ruggedness. 

#### 3.5.1. Linearity and Concentration Ranges

The linearity range was selected based on the amount of BEN and TEL in the pharmaceutical preparation. A series of seven concentrations of BEN and TEL solutions consisting of 0.5–10 µg mL^−1^ and 1–24 µg mL^−1^, respectively, were arranged separately for the second derivative method. For other methods, seven solutions in the range of 0.5–10 µg mL^−1^ for BEN and 1–30 µg mL^−1^ for TEL were analyzed in triplicate. From the generated linearity curve, regression equations and regression coefficients were generated (Table 1).

#### 3.5.2. Limit of Detection (LOD) and the Quantification (LOQ)

The limit of detection (LOD) and the limit of quantification (LOQ) represents the sensitivity of the analytical methods. LOD and LOQ were computed from the residual value (σ) of the calibration curve using 3.3 σ/s and 10 σ/s, respectively, where σ is the residual value of linearity curves of BEN and TEL and s is the slope of the corresponding calibration curve. The observed LOD and LOQ are sufficient to quantify both the analytes in the formulation ratio (Table 1).

#### 3.5.3. Accuracy and Precision

The repeatability of the anticipated procedure was assessed by performing within-day precision and accuracy by analyzing analytes at three different levels (low, medium, and high) within the linearity range. The same solutions were assayed for three continuous days for between-day precision and accuracy. The low percent relative standard deviation of 0.67–1.796 for BEN and 0.765–1.622 for TEL (Table 1) confirmed the precision of the proposed derivative UV spectroscopic procedures. Accuracy was expressed as percent recovery and percent relative error. The accuracy results tabulated in Table 1 showed 98.37%–100.62% for BEN and 98.44%–100.76% recovery for TEL, along with less than 2 percent relative error, confirming the accuracy.

#### 3.5.4. Selectivity

A series of BEN and TEL solutions having different ratios of analytes were analyzed in triplicate using the proposed UV spectroscopic procedures. The concentration was selected based on the amount of both the analytes in the pharmaceutical preparations. The selectivity was confirmed by the percentage recovery, %RSD, and %RE tabulated in Table 2. The good % recovery of around 100% along with low %RSD and %RE confirmed the accuracy and precision of the procedures. 

#### 3.5.5. Ruggedness

The consistency of the results was determined by analyzing the formulation by two different analysts on two different days. The ruggedness was expressed in terms of percentage assay and %RSD values (Table 3). The percentage assay was found to be 98.27–100.78% and 98.59–100.28% for BEN by analyst 1 and analyst 2, respectively. The percentage assay for TEL was 98.06–100.12% and 98.62–101.04% by analyst 1 and analyst 2, respectively. The %RSD values are between 0.47% and 1.72%, which is well within the acceptable criteria (<2%). 

### 3.6. Application of Proposed Methods for Assay of Solid Dosage Form

Sample solutions of BEN and TEL formulation consisting of (4 mg + 40 mg and 4 mg + 80 mg) were diluted with the 10% *v*/*v* ethanol-water mixture. The solutions were analyzed in triplicate for the assay (Supplementary File S1). Additionally, the standard addition technique was implemented to confirm the percentage recovery from the formulation and to know the interference, if any, from the formulation excipients. The percentage purity determined from the formulation solution showed the same amount as mentioned in the label (Table 4). Furthermore, excellent percentage recovery and low percentage RSD determined by the standard addition method confirmed the selectivity of the method along with accuracy and precision.

### 3.7. Evaluation of Environmental Sustainability

#### 3.7.1. Analytical GREEnness Metric Approach (AGREE)

**Figure 6:**
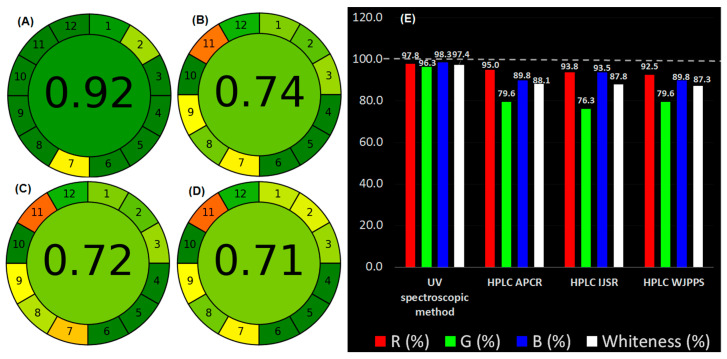
AGREE score for UV spectrophotometric method (**A**) and HPLC methods (**B**–**D**). Comparison of whiteness score between UV spectrophotometric method and HPLC methods (**E**)—HPLC IJSR [32], HPLC WJPPS [33], and HPLC APCR [34].

AGREE is a customizable eco-friendly rating tool for analytical methodologies established on green analytical chemistry principles [46]. Each principle’s contribution is converted to a unique estimate in the range of 0–1, and the overall greenness nature of the analytical method is depicted as a symbolic circle with each parameter’s score and the overall response of the technique in the middle of the AGREE symbolic circle. A total score of 1 along with dark green color represents the eco-friendly nature of the analytical procedure. The AGREE response for the anticipated UV spectroscopic procedures confirmed the method’s greenness with a score of 0.92 (Figure 6A), which is better than the three HPLC methods given (0.74, 0.72, and 0.71) (Figure 6C,D). It is clear from the AGREE score that UV spectroscopic methods are better than HPLC in terms of greenness due to the use of safer solvents compared to the toxic solvents used in HPLC methods, in addition to the high amount of waste generated in the HPLC methods (Supplementary File S2).

#### 3.7.2. White Analytical Chemistry Assessment Technique (12 RGB Design)

The recently created white analytical chemistry (WAC) tool for evaluating the greenness of analytical methods was used to judge the suggested analytical procedure’s greenness and compare it to previously published methods [47]. All 12 green analytical principles, as well as the usability and cost-effectiveness of the analytical methods, are evaluated by WAC. WAC is constructed on the RGB (red, green, and blue) design, which consists of 12 parameters. Parameters are equally distributed among RGB color groups. The red group assesses the analytical method’s applicability, correctness, reproducibility, and sensitivity; the green group assesses parameters associated with environmental protection, such as nature of reagent, the number and quantity of solvents used, waste produced during the entire development, energy consumed, and total environmental impact. The third blue group evaluates the economy, time, least practicable necessities, and easy operational capacity. WAC is a basic quantitative evaluation tool that calculates the whiteness of the analytical approach to determine sustainability. The developed UV spectroscopic method is white with a score of 97.4 compared to reported HPLC methods [32,33,34] (Figure 6E, Supplementary File S3). The presented UV spectroscopic method had better sensitivity, and safety, and was environmentally friendly, because the scaling factor was used to boost sensitivity and nontoxic dilute ethanol was used as a solvent. 

## 4. Conclusions

The concurrent quantification of BEN and TEL in fixed-dose combinations was proposed in this study using a highly efficient, practical, and environmentally friendly derivative UV spectroscopic approach. This is the first UV spectroscopic method to determine telmisartan and benidipine simultaneously in the formulation. The scaling factors of 100 and 200 were used for the quantification of BEN in the presence of a large amount of TEL. Furthermore, as compared to HPLC procedures, manipulation of UV spectra generated using a non-toxic solvent made the proposed methods green and white, saving the environment. Furthermore, the procedures were effectively applied to quantify BEN and TEL from formulations and laboratory mixed solutions with a good % recovery of 98.27–101.05% and 98.85–100.16%, respectively, for both the analytes. The proposed approaches were shown to have advantages such as a broad linear range, high precision, and environmental friendliness. As a result, this work establishes novel procedures for detecting analytes in formulations with large differences in concentration. Further, the UV spectroscopic method has limitations in terms of specificity, because impurities and degradation products cannot be identified and quantified.

## Figures and Tables

**Table 1:** Validation parameter results of the proposed spectroscopic methods for the simultaneous determination of BEN and TEL.

Validation Parameters	Benidipine				Telmisartan			
	SDM	RAD	RFD	CSM	SDM	RAD	RFD	CSM
Wavelength (nm)	239.8	360.8–304.2	344.0	238.2	233.1	305.4–244.5	318.6	299.5
Linearity range (µg mL^−1^)	0.5–10	0.5–10	0.5–10	0.5–10	1–24	1–30	1–30	1–30
Slope	0.0509	1.0784	4.5038	0.0445	0.1068	1.5785	8.3934	0.0518
Intercept	−0.0087	−0.0941	−0.4644	−0.0062	0.0149	0.6598	2.6796	−0.0089
Regression coefficient (r^2^)	0.9998	0.9998	0.9999	0.9997	0.9998	0.9997	0.9998	0.9995
LOD (µg mL^−1^)	0.121	0.088	0.099	0.139	0.288	0.287	0.256	0.268
LOQ (µg mL^−1^)	0.402	0.293	0.332	0.465	0.962	0.951	0.801	0.945
Accuracy (Mean % ± RSE)	99.61 ± 1.47	99.31 ± 0.94	100.62 ± 1.70	98.37 ± 1.08	99.35 ± 0.89	98.44 ± 0.83	99.27 ± 1.58	100.76 ± 0.67
Precision (%RSD)								
Intraday	0.864	1.247	0.679	0.758	1.471	0.947	1.622	0.765
Interday	1.408	1.353	0.964	1.796	1.592	1.203	1.493	1.531

RSE: relative standard error; RSD: relative standard deviation.

**Table 2:** Assay results of the laboratory mixed solutions of BEN and TEL.

Laboratory Prepared Mixture (µg mL^−1^)		Benidipine (% Recovery)				Telmisartan (% Recovery)			
BEN	TEL	SDM	RAD	RFD	CSM	SDM	RAD	RFD	CSM
1	20	100.45	100.62	98.48	99.63	98.76	100.54	98.47	101.34
5	20	99.54	98.34	99.57	100.92	99.34	99.33	99.48	98.47
10	10	100.57	100.86	98.37	98.83	98.81	99.07	99.62	100.62
0.5	5	99.06	100.92	100.65	100.75	100.83	101.73	98.24	99.54
2	24	99.72	100.04	99.53	100.17	99.66	100.04	98.42	99.54
Across Mean		99.87	100.16	99.32	100.06	99.48	100.14	98.85	99.90
Standard Deviation		0.76	0.49	1.14	0.98	1.01	1.35	0.75	0.62

**Table 3:** Ruggedness results.

Analyst	Benidipine (Mean % ± %RSD)				Telmisartan (Mean % ± %RSD)			
	SDM	RAD	RFD	CSM	SDM	RAD	RFD	CSM
Analyst 1	99.45 ± 0.95	100.78 ± 1.58	98.27 ± 0.67	99.43 ± 0.72	100.12 ± 1.47	99.64 ± 1.08	98.06 ± 0.47	99.11 ± 0.93
Analyst 2	99.07 ± 0.94	98.59 ± 0.85	99.66 ± 0.82	100.28 ± 0.95	99.53 ± 1.25	98.62 ± 1.06	99.43 ± 1.72	101.04 ± 1.23

**Table 4:** Assay results of the formulation and the standard addition method results.

Formulation Concentration		Benidipine (Mean% ± SD)				Telmisartan (Mean% ± SD)			
BEN	TEL	SDM	RAD	RFD	CSM	SDM	RAD	RFD	CSM
4 mg	40 mg	98.27 ± 1.34	99.07 ± 0.86	98.61 ± 1.83	98.92 ± 0.95	99.82 ± 0.66	98.87 ± 0.78	99.06 ± 0.80	98.92 ± 1.05
4 mg	80 mg	100.14 ± 0.96	100.75 ± 1.34	98.67 ± 1.06	99.55 ± 0.73	100.49 ± 1.72	99.36 ± 1.49	101.05 ± 0.79	100.67 ± 1.53
	Standard Addition Method								
Amount Added (µg mL^−1^)			Benidipine (% Recovery)			Telmisartan (% Recovery)			
1	4	99.54	98.34	99.57	100.92	99.34	99.33	99.48	98.47
2	8	100.57	100.86	98.37	98.83	98.81	99.07	100.44	100.62
3	12	99.06	100.92	100.65	100.75	100.83	101.73	100.03	99.54
Across Mean		99.72	100.04	99.53	100.17	99.66	100.04	99.98	99.54
%RSD		0.77	1.47	1.15	1.16	1.05	1.47	0.48	1.08

SD: standard deviation, %RSD: percentage relative standard deviation.

## Data Availability

The data generated during this work are included in the manuscript and its Supplementary Materials.

## Supplementary Materials

The following supporting information can be downloaded at: https://www.mdpi.com/article/10.3390/ijerph19127260/s1, Figure S1: UV absorption spectra of the formulation; Figure S2: AGREE report for UV spectroscopic and reported HPLC methods; Figure S3: Comparison of white analytical chemistry results for UV spectroscopic and reported HPLC methods; Table S1: White analytical chemistry results for UV spectroscopic and reported HPLC methods.
