# Supplementary material for: Sensitivity Enhanced Ecofriendly UV Spectrophotometric Methods for Quality Control of Telmisartan and Benidipine Formulations: Comparison of Whiteness and Greenness with HPLC Methods
Source: Int J Environ Res Public Health. 2022 Jun 14;19(12):7260. doi: 10.3390/ijerph19127260 (PMC9223904; doi:10.3390/ijerph19127260)
Supplement: Supplementary file 1 [file ijerph-19-07260-s001.zip › Supplemenatry file S1 formulation.pdf]

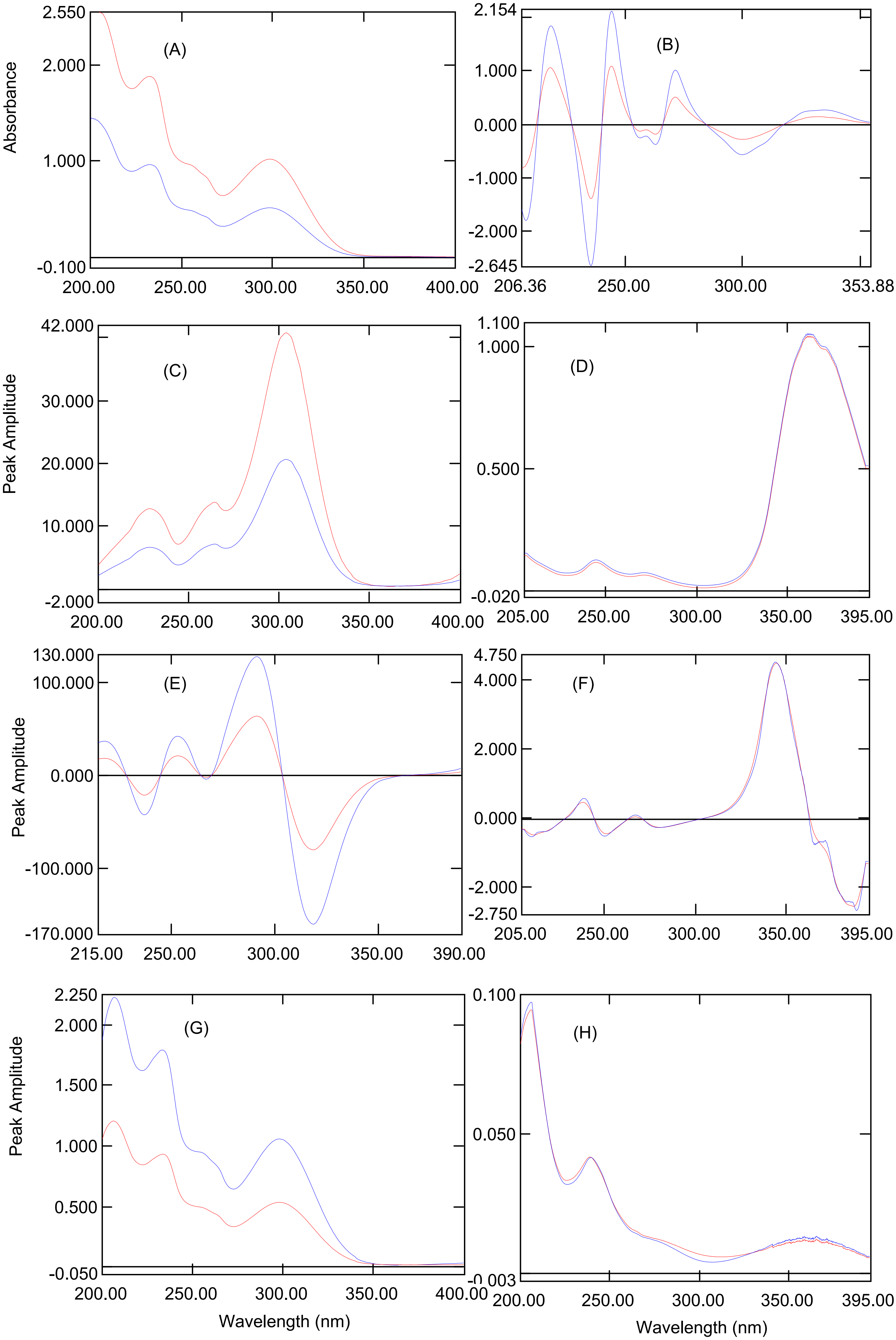

Supplementary Figures S1: UV absorption spectra of BEN and TEL formulation : Normal spectra(A) Second derivative spectra (B) Ratio derivative sepctra of TEL (C) and BEN (D) Ratio first derivative sepctra of TEL (E) and BEN (F) Zero order sepctra of TEL (G) and BEN (H)
