# Supplementary material for: Sensitivity Enhanced Ecofriendly UV Spectrophotometric Methods for Quality Control of Telmisartan and Benidipine Formulations: Comparison of Whiteness and Greenness with HPLC Methods
Source: Int J Environ Res Public Health. 2022 Jun 14;19(12):7260. doi: 10.3390/ijerph19127260 (PMC9223904; doi:10.3390/ijerph19127260)
Supplement: Supplementary file 1 [file ijerph-19-07260-s001.zip › Supplementary file S2 for AGREE.pdf]

Figure S2: Analytical Greenness report sheet

24/01/2022 17:16:48

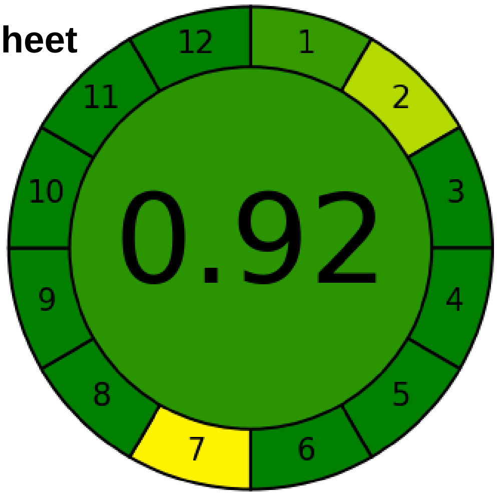

Criteria	Score	Weight
1. Direct analytical techniques should be applied to avoid sample treatment.	0.9	2
2. Minimal sample size and minimal number of samples are goals.	0.65	2
3. If possible, measurements should be performed in situ.	1.0	2
4. Integration of analytical processes and operations saves energy and reduces the use of reagents.	1.0	2
5. Automated and miniaturized methods should be selected.	1.0	2
6. Derivatization should be avoided.	1.0	2
7. Generation of a large volume of analytical waste should be avoided, and proper management of analytical waste should be provided.	0.48	2
8. Multi-analyte or multi-parameter methods are preferred versus methods using one analyte at a time.	1.0	2
9. The use of energy should be minimized.	1.0	2
10. Reagents obtained from renewable sources should be preferred.	1.0	2
11. Toxic reagents should be eliminated or replaced.	1.0	2
12. Operator's safety should be increased.	1.0	2

Analytical Greenness report sheet

25/01/2022 16:38:50

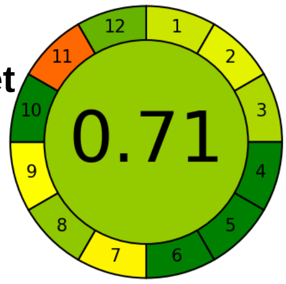

1. Sample treatment
2. Sample amount
3. Device positioning
4. Sample prep. stages
5. Automation, miniaturization
6. Derivatization
7. Waste
8. Analysis throughput
9. Energy consumption
10. Source of reagents
11. Toxicity
12. Operator's safety

Criteria	Score	Weight
1. Direct analytical techniques should be applied to avoid sample treatment.	0.6	2
2. Minimal sample size and minimal number of samples are goals.	0.55	2
3. If possible, measurements should be performed in situ.	0.66	2
4. Integration of analytical processes and operations saves energy and reduces the use of reagents.	1.0	2
5. Automated and miniaturized methods should be selected.	1.0	2
6. Derivatization should be avoided.	1.0	2
7. Generation of a large volume of analytical waste should be avoided, and proper management of analytical waste should be provided.	0.48	2
8. Multi-analyte or multi-parameter methods are preferred versus methods using one analyte at a time.	0.72	2
9. The use of energy should be minimized.	0.5	2
10. Reagents obtained from renewable sources should be preferred.	1.0	2
11. Toxic reagents should be eliminated or replaced.	0.2	2
12. Operator's safety should be increased.	0.8	2

Analytical Greenness report sheet

04/05/2022 11:47:41

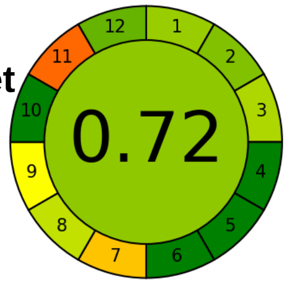

1. Sample treatment
2. Sample amount
3. Device positioning
4. Sample prep. stages
5. Automation, miniaturization
6. Derivatization
7. Waste
8. Analysis throughput
9. Energy consumption
10. Source of reagents
11. Toxicity
12. Operator's safety

Criteria	Score	Weight
1. Direct analytical techniques should be applied to avoid sample treatment.	0.7	2
2. Minimal sample size and minimal number of samples are goals.	0.75	2
3. If possible, measurements should be performed in situ.	0.66	2
4. Integration of analytical processes and operations saves energy and reduces the use of reagents.	1.0	2
5. Automated and miniaturized methods should be selected.	1.0	2
6. Derivatization should be avoided.	1.0	2
7. Generation of a large volume of analytical waste should be avoided, and proper management of analytical waste should be provided.	0.39	2
8. Multi-analyte or multi-parameter methods are preferred versus methods using one analyte at a time.	0.62	2
9. The use of energy should be minimized.	0.5	2
10. Reagents obtained from renewable sources should be preferred.	1.0	2
11. Toxic reagents should be eliminated or replaced.	0.2	2
12. Operator's safety should be increased.	0.8	2

Analytical Greenness report sheet

04/05/2022 11:51:27

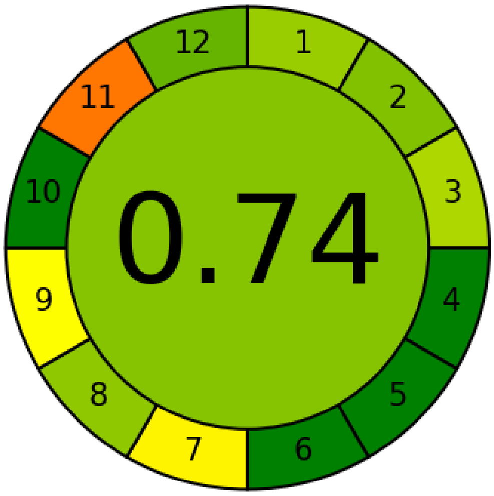

Criteria	Score	Weight
1. Direct analytical techniques should be applied to avoid sample treatment.	0.7	2
2. Minimal sample size and minimal number of samples are goals.	0.75	2
3. If possible, measurements should be performed in situ.	0.66	2
4. Integration of analytical processes and operations saves energy and reduces the use of reagents.	1.0	2
5. Automated and miniaturized methods should be selected.	1.0	2
6. Derivatization should be avoided.	1.0	2
7. Generation of a large volume of analytical waste should be avoided, and proper management of analytical waste should be provided.	0.48	2
8. Multi-analyte or multi-parameter methods are preferred versus methods using one analyte at a time.	0.72	2
9. The use of energy should be minimized.	0.5	2
10. Reagents obtained from renewable sources should be preferred.	1.0	2
11. Toxic reagents should be eliminated or replaced.	0.23	2
12. Operator's safety should be increased.	0.8	2
