# Supplementary material for: Sensitivity Enhanced Ecofriendly UV Spectrophotometric Methods for Quality Control of Telmisartan and Benidipine Formulations: Comparison of Whiteness and Greenness with HPLC Methods
Source: Int J Environ Res Public Health. 2022 Jun 14;19(12):7260. doi: 10.3390/ijerph19127260 (PMC9223904; doi:10.3390/ijerph19127260)
Supplement: Supplementary file 1 [file ijerph-19-07260-s001.zip › Supplementary file S3 for WAC.pdf]

## Supplimentary Table S1: White analytical chemistry

| RED PRINCIPLES (analytical performance) | | | R1: Scope of application | R2: LOD and LOQ | | | R3: Precision | | | R4: Accuracy | | |
|---|---|---|---|---|---|---|---|---|---|---|---|---|
| | Method number | Method name | 0-100 | LOD | LOQ | 0-100 | RSD% (repeatability) | RSD% (reproducibility) | 0-100 | Relative error (%) | Recovery (%) | 0-100 |
| | 1 | UV spectroscopic method | 95 | 0.088, 0.287 | 0.293-0.962 | 96 | 0.679-1.622 | 0.964-1.796 | 100 | 0.670-1.70 | 98.37-100.76 | 100 |
| | 2 | HPLC APCR | 100 | 0.133-0.402 | 0.458-2.589 | 80 | 0.671- 0.509 | 0.604-1.690 | 100 | ___ | 98.98-101.50 | 100 |
| | 3 | HPLC IJSR | 100 | 0.19-1.19 | 2.57-2.94 | 75 | 0.63-1.57 | 0.11-0.63 | 100 | ___ | 99.77-100.31 | 100 |
| | 4 | HPLC WJPPS | 100 | 0.147-1.306 | 0.444-3.956 | 70 | 0.44-0.55 | 0.25-0.91 | 100 | ___. | 99.45-101.17 | 100 |

| GREEN PRINCIPLES (green chemistry) | | | G1: Toxicity of reagents (impact and biodegradation) | | G2: Amount of reagents and waste | | | G3: Concumption of energy and other media | G4: Direct impacts (safety, use of animals and GMOs) | | | |
|---|---|---|---|---|---|---|---|---|---|---|---|---|
| | Method number | Method name | Total number of pictograms | 0-100 | Reagent consumption | Waste production | 0-100 | 1-100 | Occupational hazards | Safety of users (0-100) | Use of animals (0 if no, 1 if | Use of GMO (0 if no, 1 if yes) |
| | 1 | UV spectroscopic method | Safe and biodegradabl | 95 | < 10ml | < 10ml | 95 | 95 | Not Hazardus | 100 | 0 | 0 |
| | 2 | HPLC APCR | Toxic | 75 | >10 ml | >10 ml | 75 | 75 | Hazardus | 80 | 0 | 0 |
| | 3 | HPLC IJSR | Toxic | 65 | >10 ml | >10 ml | 75 | 75 | Hazardus | 70 | 0 | 0 |
| | 4 | HPLC WJPPS | Toxic | 75 | >10 ml | >10 ml | 75 | 75 | Hazardus | 80 | 0 | 0 |

| BLUE PRINCIPLES (practical side) | | | B1: Cost-efficiency | | B2: Time-efficiency | | B3: Requirements | | | B4: Operational simplicity | | |
|---|---|---|---|---|---|---|---|---|---|---|---|---|
| | Method number | Method name | Total cost USD/Sample | 0-100 | Speed of analysis /Hour | 0-100 | Sample consumption | Sample consumption (0-100) | Other needs: advanced instruments, skills, facilities (0- | Miniaturization (0-100) | Integration and automation (0-100) | Portability (0-100) |
| | 1 | UV spectroscopic method | 5 | 100 | 60 samples | 100 | in micrograms | 100 | 100 | 90 | 95 | 95 |
| | 2 | HPLC APCR | 8 | 90 | 7 samples | 75 | in micrograms | 100 | 95 | 95 | 100 | 95 |
| | 3 | HPLC IJSR | 7 | 95 | 12 samples | 85 | in micrograms | 100 | 95 | 95 | 100 | 95 |
| | 4 | HPLC WJPPS | 8 | 90 | 7 samples | 75 | in micrograms | 100 | 95 | 95 | 100 | 95 |

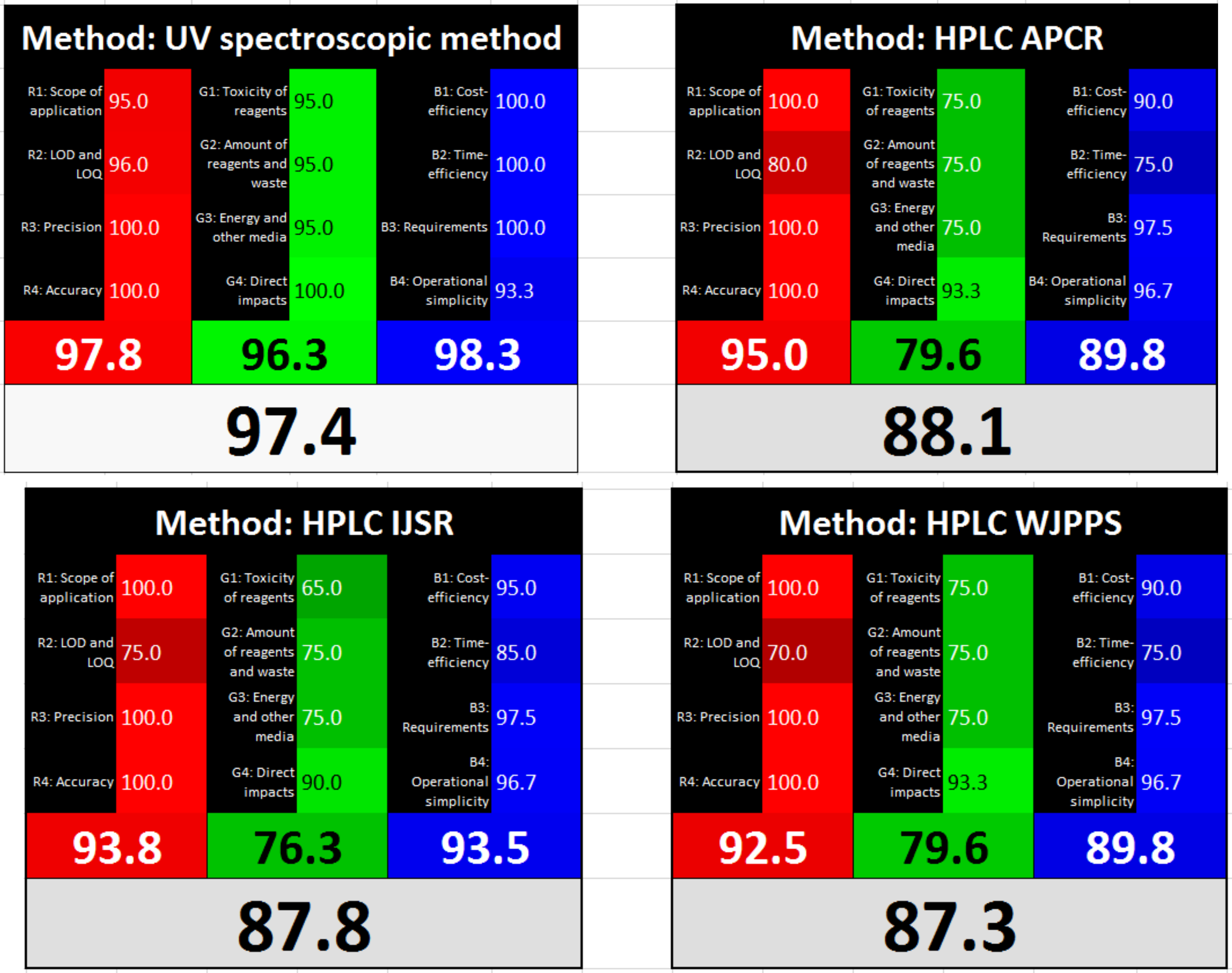


Figure S3: whiteness score of UV spectroscopic method and reported HPLC methods
